# Mediating role of plasma metabolites in the association between plasma proteins and knee osteoarthritis: An observational study

**DOI:** 10.1097/MD.0000000000050393

**Published:** 2026-08-28

**Authors:** Haibo Zheng, Ping Zeng, Xianqiu Li, Hao Ni, Longlong Wei, Xueshen Mo, Kun Hou, Xiangbin Rong

**Affiliations:** a Guangxi University of Chinese Medicine, Nanning, Guangxi Zhuang Autonomous Region, China; b The First Affiliated Hospital of Guangxi University of Chinese Medicine, Nanning, Guangxi Zhuang Autonomous Region, China.

**Keywords:** knee osteoarthritis, mediation analysis, Mendelian randomization analysis, plasma metabolites, plasma proteins

## Abstract

Knee osteoarthritis (KOA) is a prevalent degenerative joint disease with limited treatment options, and the regulatory roles of plasma proteins and metabolites in its pathogenesis remain largely unclear. To explore the causal relationships and correlations among plasma proteins, plasma metabolites, and KOA, we constructed a regulatory network integrating these 3 components. We selected a total of 4907 plasma proteins as exposure variables, 1400 plasma metabolites as mediating variables, and KOA as the outcome variable in this study. We employed inverse variance weighting as the primary analytical approach and performed pleiotropy and heterogeneity tests to guarantee the robustness of the results; additionally, we conducted reverse Mendelian randomization analysis to validate the rationality of the exclusion restriction assumption. Subsequently, we implemented mediation analysis for plasma proteins exhibiting a significant causal association with KOA. Inverse variance weighting analysis identified 2 plasma proteins with significant causal associations with KOA: insulin-like growth factor-binding protein 3 was positively correlated with KOA susceptibility (*β* = 0.065, odds ratio = 1.067, *P* = .004, 95% confidence interval: 1.021–1.115), whereas collagen type XVIII alpha 1 chain was negatively correlated with KOA (*β *= −0.111, odds ratio = 0.895, *P *= .005, 95% confidence interval: 0.830–0.966); no reverse causal relationships were detected for either protein. Mediation effect analysis revealed 20 plasma metabolites with significant mediating associations with KOA; notably, insulin-like growth factor-binding protein 3 could downregulate the levels of N-acetylmethionine and 2-O-methylascorbic acid, thereby promoting the progression of KOA. Collectively, the identified causal associations and mediating effects provide a theoretical foundation and research orientation for the development of KOA therapeutic strategies targeting plasma proteins.

## 1. Introduction

Osteoarthritis (OA) is the most prevalent joint degenerative disease worldwide, which severely impairs the health of middle-aged and elderly populations.^[1]^ Its incidence rises significantly with age, and it ranks among the leading causes of chronic pain and physical disability. The global disease burden of OA is substantial^[2]^: a meta-analysis involving over 10 million participants demonstrated that the overall prevalence rate reaches 16.0% in individuals aged 15 years and above and climbs to 22.9% in those aged 40 years and older. Notably, OA primarily affects weight-bearing or frequently mobile joints such as the knees, hips, and hands. The core pathological feature is the progressive degeneration of articular cartilage, accompanied by subchondral bone sclerosis and remodeling, osteophyte formation, and synovial inflammation, which ultimately lead to joint pain and functional impairment.^[3]^ Currently, diverse clinical treatment modalities are available yet with notable limitations: non-pharmacological therapies fail to halt disease progression; pharmacological treatments (e.g., nonsteroidal anti-inflammatory drugs and glucocorticoids) are restricted by their adverse effects; and total joint replacement, the standard treatment for end-stage OA, is associated with considerable surgical trauma, high medical costs, and inherent operative risks. Therefore, there is an urgent and critical need to develop more effective strategies for the prevention and treatment of OA.

In recent years, with the in-depth exploration of the pathogenesis of OA, plasma proteins have emerged as a potential research hotspot. As important functional molecules in the blood, they not only participate in various physiological processes such as substance transport, immune regulation, and coagulation and hemostasis, but also indirectly affect the homeostasis of the joint microenvironment by regulating the production, transport, and transformation of plasma metabolites, thereby providing a novel perspective for understanding the systemic regulatory mechanisms of OA.^[4,5]^ For instance, through large-scale proteomic analysis of blood samples from 45,307 participants in the United Kingdom Biobank, Kang ZJ et al^[6]^ identified that multiple plasma proteins including collagen type IX alpha‑1 chain and cartilage acidic protein 1 were closely linked with the risk of OA, furnishing crucial clues for the early detection and warning of the disease. Goldring SR et al^[7]^ characterized a variety of plasma proteins in the synovial fluid of knee osteoarthritis (KOA) patients and demonstrated that several of these proteins could activate macrophages via the innate immune receptor toll-like receptor 4, elaborating a novel mechanism that triggers synovial inflammation in KOA patients. Another study by Benson MD et al^[8]^ which analyzed 3626 plasma samples, revealed that 1265 proteins had 171,800 significant associations with 365 metabolites, providing evidence for clarifying the regulatory effect of plasma proteins on plasma metabolites and their impact on the homeostasis of the joint microenvironment. Plasma proteins are involved in the pathological process of OA by dynamically regulating the homeostasis of the joint microenvironment: on the one hand, inflammation-related plasma proteins (e.g., C-reactive protein and tumor necrosis factor-α) can activate the synovial inflammatory cascade and promote the catabolism of chondrocytes; on the other hand, the imbalance of matrix metabolism-related proteins (e.g., matrix metalloproteinases and their inhibitors) can accelerate the degradation of cartilage matrix, while the abnormality of coagulation-fibrinolysis system proteins is associated with impaired repair of subchondral bone microdamage. The changes in the spectrum of these plasma proteins are not only biomarkers of the pathological state of KOA, but also serve as key hubs connecting systemic metabolic disorders and local joint degeneration by mediating signal crosstalk between the blood circulation and the joint microenvironment. Although the above studies have investigated the association between plasma proteins and KOA from different perspectives, encompassing large-scale risk association analyses, local joint inflammatory mechanisms, and protein–metabolite correlation networks, most have focused on a single omics approach or localized pathological processes.^[9,10]^ In contrast, building upon existing research, the present study focuses on systematic integration and in-depth analysis. Based on 4907 plasma proteins and 1400 plasma metabolites, this study specifically explores the mediating role of plasma metabolites in the association between plasma proteins and KOA. Furthermore, it elucidates the intrinsic relationships and potential regulatory mechanisms among plasma proteins, plasma metabolites, and joint microenvironment homeostasis, aiming to provide more comprehensive research insights and theoretical references for the precise prevention and treatment of KOA.

In summary, the core aims of this study are clearly stated as follows: to clarify the intrinsic association characteristics between plasma proteins and KOA, and to reveal the mediating mechanism of plasma metabolites in their interaction; to screen for key plasma proteins and metabolites associated with KOA pathogenesis, and to elaborate on their specific pathways in regulating joint microenvironmental homeostasis and participating in the disease’s pathological process; and ultimately to provide an experimental basis and theoretical support for unraveling the pathogenesis of KOA, identifying new therapeutic targets, and formulating precise prevention and treatment strategies. On this basis, this study not only provides a multi-dimensional, novel perspective for KOA research but also aligns with the core requirements of modern preventive medicine, which is characterized by precision early warning, targeted intervention, and population-based prevention and control, thus holding substantial theoretical and practical significance.

## 2. Materials and methods

### 2.1. Study design

**Figure 1.**
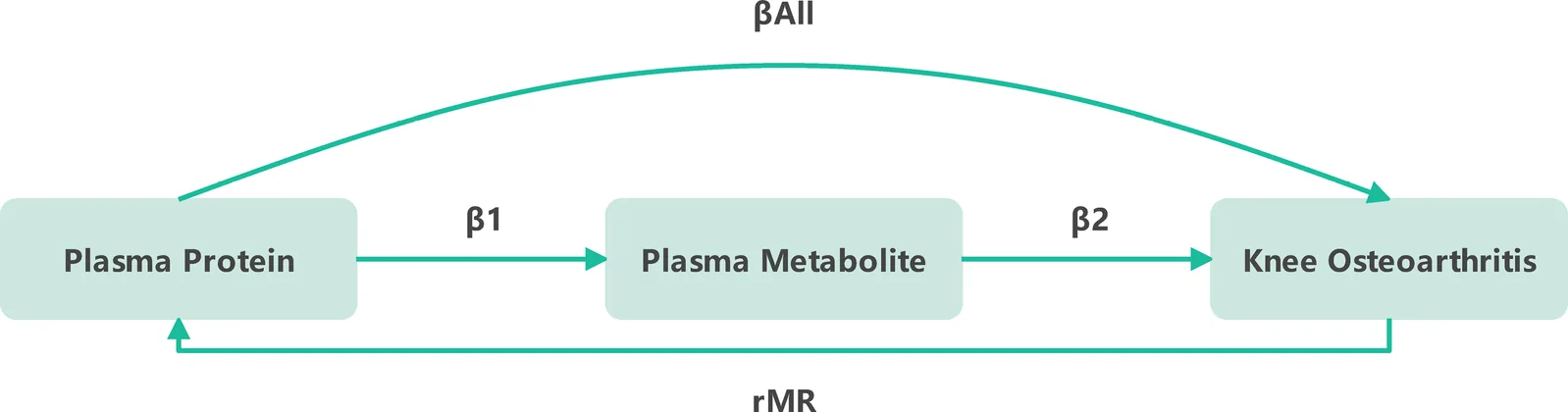
Schematic of the mediating effect framework between plasma proteins, plasma metabolites, and KOA. This diagram illustrates the causal relationships analyzed in this study: β_1_ denotes the effect of plasma protein on plasma metabolite, β_2_ denotes the effect of plasma metabolite on KOA, β_all_ denotes the total effect of plasma protein on KOA, and rMR was used to verify reverse causality in the pathway. KOA = knee osteoarthritis, rMR = reverse Mendelian randomization.

The flowchart depicting the overall framework of this study design is presented in Figure 1. Mendelian randomization (MR) analysis was performed using genome-wide association study (GWAS) summary-level data encompassing 4907 plasma proteins, 1400 plasma metabolites, and KOA to evaluate the causal associations among these 3 components. MR analysis utilizes genetic variants to proxy risk factors, and valid instrumental variables (IVs) for causal inference must satisfy 3 key assumptions: relevance assumption: IVs are strongly associated with the exposure of interest (plasma proteins/metabolites in this study); independence assumption: IVs are independent of potential confounders between the exposure and the outcome (KOA); exclusion restriction assumption: IVs can affect the outcome only through the exposure.

### 2.2. Data sources

Plasma protein data were obtained from the deCODE genetics database in Iceland. This database focuses on genetic research and provides large-scale datasets, including plasma proteomics data, to facilitate the elucidation of gene-disease associations. A total of 40,004 Icelanders were recruited between August 2000 and January 2019 (52% from the Icelandic Cancer Project, 48% from deCODE-related programs). After screening (Icelandic nationality, informed consent, qualified genetic and protein data; excluding poor-quality samples and aptamers), 35,559 participants were finally included. Plasma samples were collected using ethylenediaminetetraacetic acid tubes and processed subsequently, with proteins detected using SomaScan v4 technology (retaining 4907 aptamers). Genetic data were acquired through whole-genome sequencing and chip genotyping.^[11]^ Plasma metabolite data were obtained from published literature.^[12]^ The study enrolled European participants aged 45 to 85 years. Unrelated individuals with available genome-wide genotyping and plasma metabolite data were included, while those with familial relationships or low-quality data were excluded. After detection and strict quality control, 1091 plasma metabolites were retained, and 309 metabolite ratios were constructed for association analysis. KOA data were obtained from the Integrative Epidemiology Unit Open GWAS database (ID: ebi-a-GCST007092). The dataset included 417,596 European ancestry samples, and the genome build was Genome Reference Consortium Human Build 37, also known as Human Genome Build 19. Developed by the United Kingdom Medical Research Council Integrative Epidemiology Unit, this database aims to collect high-quality GWAS summary statistics for genetic epidemiological research. The GWAS summary statistics for KOA have been fully validated and can be directly used for MR analysis. This study conducted a secondary data analysis using freely available data from public databases. All data included in this study have received ethical approval from their respective original institutions. Therefore, no additional ethical review was required for this secondary analysis.

### 2.3. Experimental methods

#### 2.3.1. Instrument selection

For the MR analysis, single nucleotide polymorphisms (SNPs) strongly associated with the phenotypes of specific plasma proteins (*P* < 5e−08), plasma metabolites (*P* < 1e−05), and KOA (*P* < 5e−08) were selected as IVs. To evaluate the strength of IVs (IV) and mitigate weak IV bias, the *F* statistic was calculated for each IV, with an *F* statistic > 10 considered as the threshold for excluding weak instruments. SNPs with *F* > 10 were considered legitimate IVs, thus mitigating weak instrument bias. *R*^2^ is determined using the formula: $R^{2}=\frac{\overset{2}{\underset{exposure}{\beta }}}{\overset{2}{\underset{exposure}{\beta }}+\overset{2}{\underset{exposure}{se}}\cdot {samplesize}_{exposure}}$, and the *F* statistic is calculated by $F=\frac{R^{2}\cdot \left({samplesize}_{exposure}-2\right)}{1-R^{2}}$. Here, $\beta_{exposure}$ represents the regression coefficient, $Se_{exposure}$ is the standard error, and ${\;samplesize}_{exposure}$ is the sample size.^[13]^ This study screened eligible instrumental variable SNPs for 3 exposures: 31,315 SNPs for plasma protein phenotypes (Supplementary File, Supplemental Digital Content 1 1), 34,843 SNPs for plasma metabolites (Supplementary File, Supplemental Digital Content 1 2), and 26 SNPs for KOA (Supplementary File, Supplemental Digital Content 1 3). All these SNPs were evaluated based on certain criteria and subsequently utilized in MR analysis. Ultimately, allele data were compared and rectified by means of the SNP2eaf function (in the TwoSampleMR package) to mitigate the impact of alleles on the results and to guarantee the precise calculation of effective allele frequencies.

#### 2.3.2. Statistical analyses

All analytical modules in this study were implemented in R software (Version 4.4.1; R Core Team, https://www.r-project.org/), with key analyses performed using the TwoSampleMR package developed by the MRC Integrative Epidemiology Unit for MR. Heterogeneity tests^[14]^ were performed using the MR-Egger and inverse variance weighting (IVW) methods, with Cochran *Q* statistic applied for quantification to assess the consistency of causal effect estimates across IVs. Pleiotropy was assessed via MR-Egger regression and the MR-pleiotropy residual sum and outlier test to exclude the potential bias caused by horizontal pleiotropy on causal effect estimates.^[15]^ Leave-one-out sensitivity analysis was conducted by sequentially excluding each individual IV and re-performing the analysis to determine whether the causal association was driven by any single SNP and to verify the robustness of the causal estimates.

#### 2.3.3. MR analysis

MR analysis was performed using the TwoSampleMR package in R. Five analytical methods were employed, namely MR-Egger regression, weighted median, IVW, simple mode, and weighted mode.^[16]^ The IVW method was designated as the sole filtering approach to identify exposures with strong associations with the outcome, with the following filtering criteria set: for the association from plasma proteins to KOA and from plasma metabolites to KOA, a *P* value < .01 was required; the direction of the odds ratio (OR) was consistent with that of the beta value (*β*); the absence of horizontal pleiotropy (MR-Egger intercept *P* > .05) and heterogeneity (Cochran *Q* test *P* > .05) was confirmed.

#### 2.3.4. Reverse MR analysis

The methods and principles of reverse MR analysis were consistent with those of the forward MR analysis. Plasma proteins filtered by the IVW method were designated as the outcome, while KOA was defined as the exposure. The *P* value derived from the IVW causal effect test was used as the reference standard; a *P* value > .05 indicated a negative result, suggesting the absence of a reverse causal relationship.

#### 2.3.5. Mediation analysis

Plasma proteins and plasma metabolites strongly associated with the outcome after IVW-based filtering were subjected to further MR analysis. The regression coefficient (*β*, effect size), which reflects the direction and strength of the association between the exposure and the outcome in the MR analysis results, was adopted as the reference standard.^[17]^ The result of the plasma protein-to-plasma metabolite association was denoted as *β*_1_, and that of the plasma metabolite-to-KOA association as *β*_2_; the result of the plasma protein-to-KOA association was defined as the total effect (*β*_all_). The mediating effect size was calculated as *β*_1_ × *β*_2_, and the proportion of the mediating effect was determined by dividing the mediating effect size by the total effect value (*β*All).

## 3. Results

### 3.1. Results of stepwise forward MR analysis and statistical analysis

**Figure 2.**
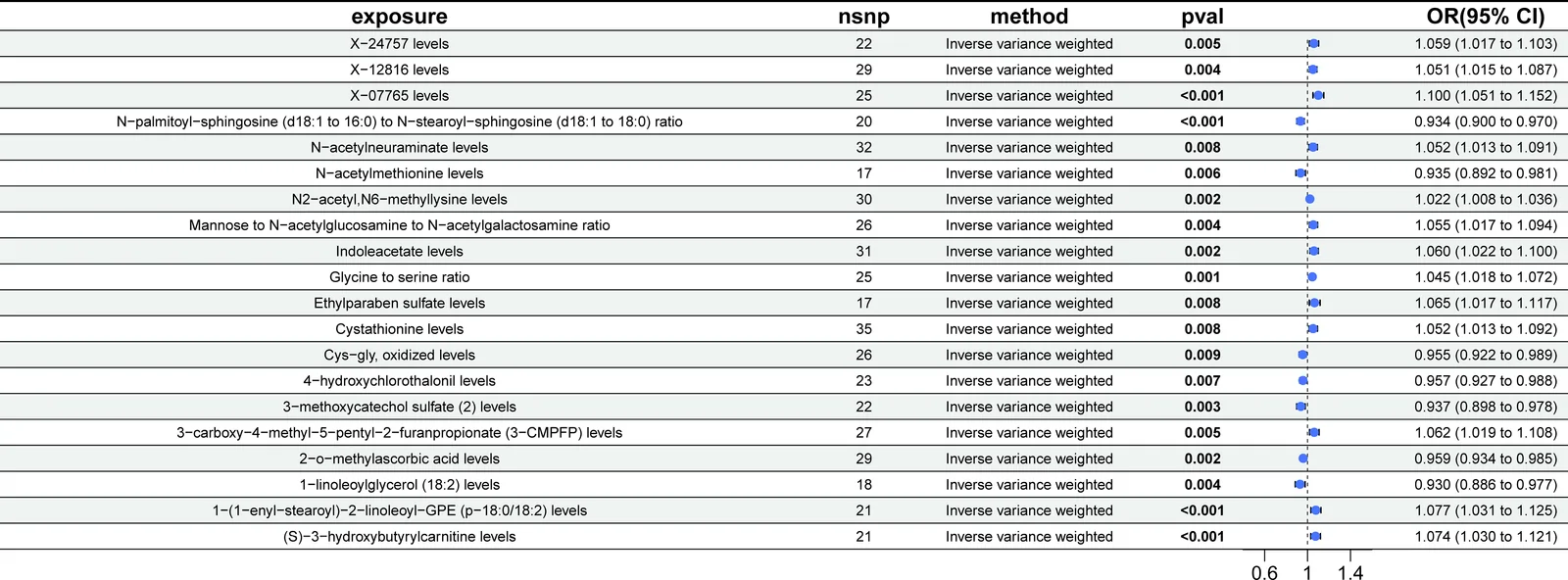
Forest plot visualization of 20 plasma metabolites. This figure shows the exposure factors, number of IV SNPs (nSNP), inverse variance weighting method, *P* values (*P*val), and OR with 95% CI of plasma metabolites strongly associated with KOA. CI = confidence interval, KOA = knee osteoarthritis, OR = odds ratios, SNP = single nucleotide polymorphism.

**Figure 3.**
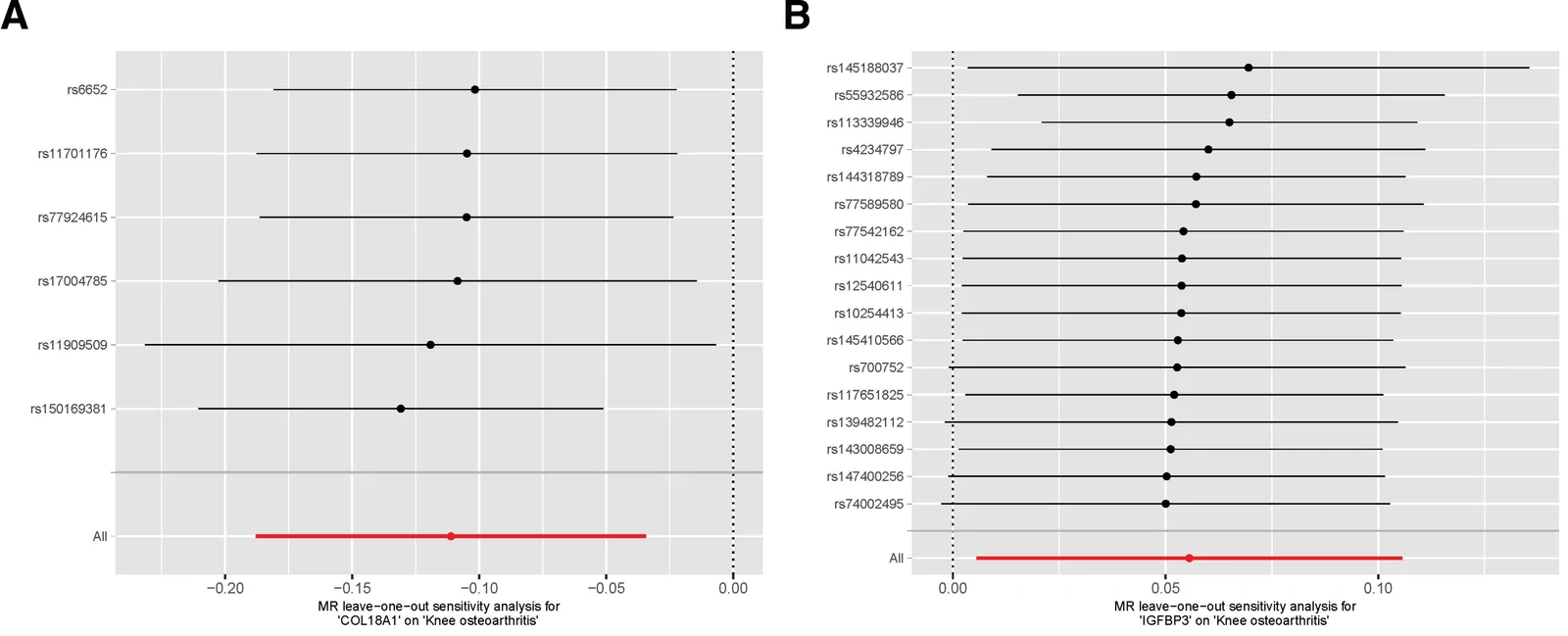
Leave-one-out analysis plots of 2 plasma proteins. Panels (A) and (B) represent the MR leave-one-out sensitivity analysis plots of COL18A1 and IGFBP3 proteins, respectively. These plots show the changes in effect sizes after sequentially removing each instrumental variable SNP, with the red horizontal line indicating the effect estimate when all SNPs are included. COL18A1 = collagen type XVIII alpha 1 chain, IGFBP3 = insulin-like growth factor-binding protein 3, KOA = knee osteoarthritis, SNP = single nucleotide polymorphism.

Through the IVW method and predefined filtering criteria, collagen type XVIII alpha 1 chain (COL18A1) was identified to be negatively correlated with KOA progression (*β* = −0.111, OR = 0.895, *P* = .005, 95% confidence interval: 0.895–0.966); in contrast, insulin-like growth factor-binding protein 3 (IGFBP3) was found to be positively correlated with KOA progression (*β* = 0.065, OR = 1.067, *P* = .004, 95% confidence interval: 1.021–1.115). Meanwhile, 20 plasma metabolites were detected to be associated with KOA, as illustrated in Figure 2. Statistical analysis results demonstrated that the *P* values corresponding to the MR-Egger intercept in pleiotropy tests were all > .05, and no biased SNPs were detected by the MR-pleiotropy residual sum and outlier test (see Table 1). For heterogeneity tests, the *P* values corresponding to Cochran *Q* statistic derived from the MR-Egger and IVW methods were both > .05, indicating the absence of heterogeneity (see Table 2). The leave-one-out analysis plots showed that the exclusion of any single SNP did not exert a substantial impact on the IVW-derived effect estimates, which verified the robustness of the identified causal relationships (see Fig. 3).

**Table 1:** Pleiotropy test results of 2 plasma proteins and KOA.

Exposure	Outcome	Pleiotropy test						
		MR-Egger regression egger_intercept	SE	*P*	MR-PRESSO MR-PRESSO_Global MR-PRESSO_Outlier			
					RSSobs	*P* value	RSSobs	*P*
COL18A1	KOA	−0.013	0.009	.238	5.321	.616	NA	NA
IGFBP3	KOA	0.001	0.005	.897	27.209	.119	NA	NA

The exposures were 2 plasma proteins, COL18A1 and IGFBP3, and the outcome was KOA. Egger_intercept is the intercept of MR-Egger regression. MR-PRESSO_global: global test statistic; MR-PRESSO_outlier: outlier test.

COL18A1 = collagen type XVIII alpha 1 chain, IGFBP3 = insulin-like growth factor-binding protein 3, KOA = knee osteoarthritis, MR = Mendelian randomization, PRESSO = pleiotropy residual sum and outlier, RSSobs = observed residual sum of squares, SE = standard error.

**Table 2:** Heterogeneity test results of 2 plasma proteins and KOA.

Exposure	Outcome	Heterogeneity test					
		MR-Egger			IVW		
		*Q*	*Q*_df	*Q (P*)	*Q*	*Q*_df	*Q (P*)
COL18A1	KOA	2.629	4	.622	4.549	5	.473
IGFBP3	KOA	17.613	14	.225	17.635	15	.282

*Q* refers to the test statistic; *Q*_df refers to the degrees of freedom of the *Q* statistic; *Q*_P refers to the *P* value corresponding to the *Q* statistic.

COL18A1 = collagen type XVIII alpha 1 chain, IGFBP3 = insulin-like growth factor-binding protein 3, IVW = inverse variance weighted, KOA = knee osteoarthritis, MR = Mendelian randomization.

### 3.2. Results of reverse MR analysis

The IVW method yielded the following results: COL18A1 (*P* = .085) and IGFBP3 (*P* = .285). Similar nonsignificant results (*P* > .05) were obtained using the other 4 analytical approaches, namely MR-Egger regression, weighted median, simple mode, and weighted mode, as presented in Table 3.

**Table 3:** Results of rMR analysis between 2 plasma proteins and KOA.

Exposure	Outcome	Methods	nSNP	*P*val
KOA	COL18A1	MR-Egger	23	.235
		Weighted median	23	.217
		IVW	23	.085
		Simple mode	23	.864
		Weighted mode	23	.670
KOA	IGFBP3	MR-Egger	24	.961
		Weighted median	24	.243
		IVW	24	.285
		Simple mode	24	.351
		Weighted mode	24	.293

The exposure was KOA; the outcome was 2 plasma proteins, COL18A1 and IGFBP3. COL18A1 = collagen type XVIII alpha 1 chain, IGFBP3 = insulin-like growth factor-binding protein 3, IVW = inverse variance weighted, KOA = knee osteoarthritis, MR = Mendelian randomization, nSNP = number of single nucleotide polymorphisms, rMR = reverse Mendelian randomization.

### 3.3. Results of the intermediary effects

**Figure 4.**
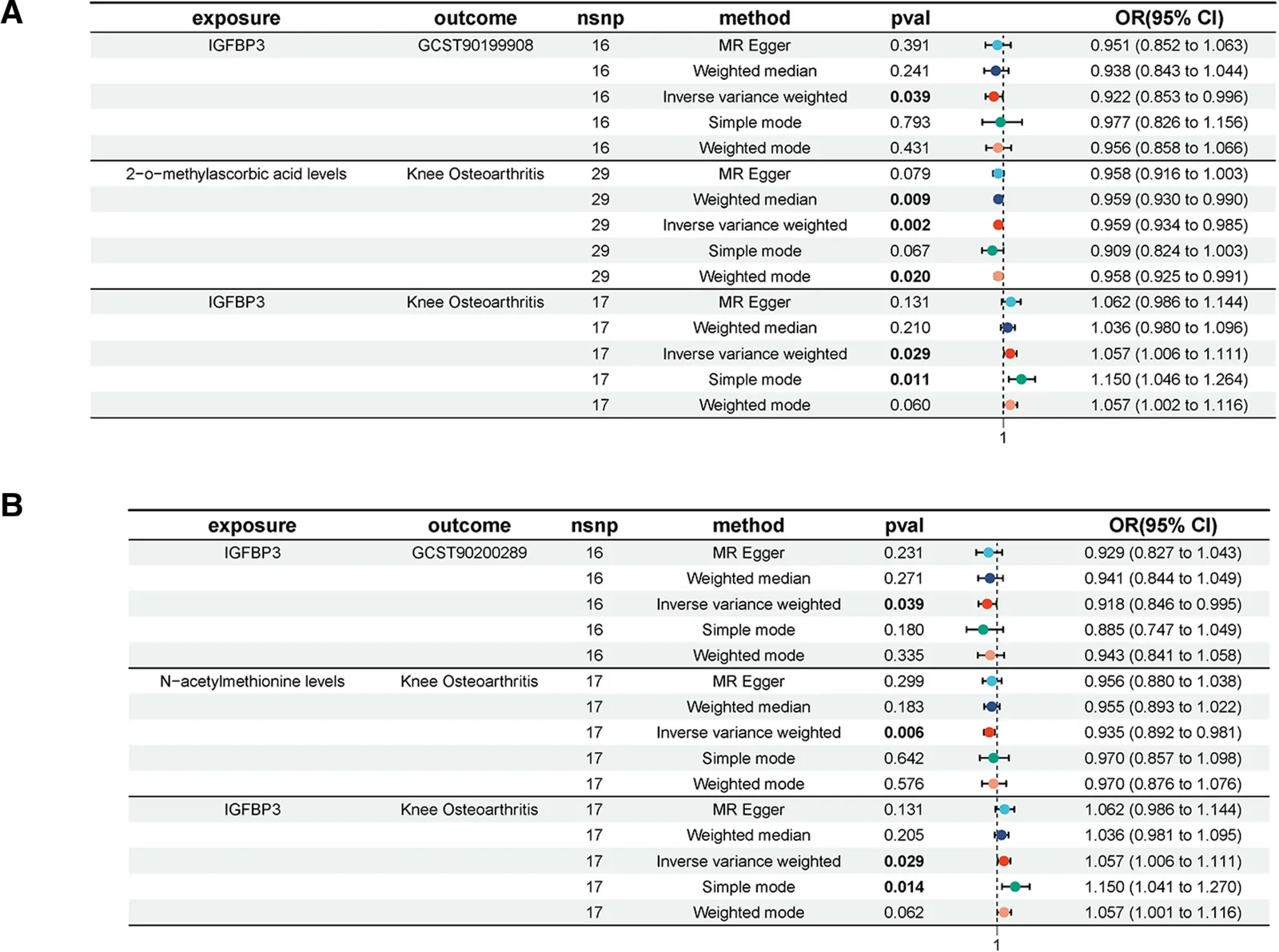
Mediation MR analysis plot of IGFBP3. (A) Results of MR analysis for the regulation of 2-O-methylascorbic acid by IGFBP3; (B) results of MR analysis for the regulation of N-acetylmethionine by IGFBP3. CI = confidence interval, IGFBP3 = insulin-like growth factor-binding protein 3, KOA = knee osteoarthritis, MR = Mendelian randomization, OR = odds ratios, SNP = single nucleotide polymorphism.

Mediation effect analysis was performed on the 20 plasma metabolites, which revealed that the levels of 2-O-methylascorbic acid (*β*_1_ = −0.081, *β*_2_ = −0.042, mediating effect = 0.003, *β*_all_ = 0.056, representing 5.40% of the total effect) and N-acetylmethionine (NAM) (*β*_1_ = −0.086, *β*_2_ = −0.067, mediating effect = 0.006, *β*_all_ = 0.056, representing 10.70% of the total effect) mediated the causal association between human IGFBP3 and KOA. Furthermore, elevated levels of these 2 plasma metabolites were found to attenuate the progression of KOA, as illustrated in Figure 4.

## 4. Discussion

Our study identified that COL18A1, encoded by the COL18A1 gene, attenuates the progression of KOA. Relevant studies have indicated that^[18]^ COL18A1 plays a key role in regulating extracellular matrix (ECM) homeostasis and the transmission of mechanical signals between cells and the matrix. In KOA, abnormal mechanical loading and inflammatory responses disrupt ECM homeostasis,^[19]^ while COL18A1 may help maintain the structural integrity of basement membranes and collagen-containing ECM, buffer the direct mechanical stress-induced damage to chondrocytes, and reduce the abnormal accumulation of inflammatory factors in the joint cavity, thereby delaying disease progression. From a molecular functional perspective, COL18A1 can also specifically bind to cell surface receptors such as integrins (e.g., α10*β*1 integrin), activating downstream signaling pathways including phosphatidylinositol 3‑kinase (PI3K)/protein kinase B (AKT) and mitogen‑activated protein kinase, which in turn inhibit chondrocyte apoptosis and reduce synovial inflammation. These PI3K/AKT and mitogen‑activated protein kinase pathways regulate cell proliferation, migration, and differentiation, which not only enhance cell adhesion capacity to reduce chondrocyte detachment caused by ECM degradation, but also improve cellular tolerance to mechanical stress by modulating cytoskeletal dynamics. This mechanism is supported by multiple in vitro and in vivo studies. For instance, Hodgkinson T et al found that^[20]^ mechanical signals regulate chondrocyte ECM metabolism via integrins and mechanosensitive ion channels, and that abnormal mechanical loading activates the rat sarcoma/rapidly accelerated fibrosarcoma/mitogen‑activated protein kinase kinase/extracellular signal‑regulated kinase pathway to promote ECM degradation. Jin HF et al^[21]^ concluded in their review that the binding of α10*β*1 integrin to COL18A1 protects the ECM by regulating cell adhesion and mechanical signal transmission. Fukai N et al^[22]^ indirectly confirmed the role of COL18A1 in maintaining ECM structure through the observation that COL18A1-knockout mice exhibited basement membrane disruption and vascular abnormalities. The present study for the first time clarifies the causal inhibitory effect of COL18A1 on KOA progression, and links its underlying mechanism to ECM homeostasis, mechanical signal transduction, and integrin-mediated signaling pathways. These findings provide a novel molecular target perspective for research into the pathological mechanisms of osteoarthritis, as well as a potential universal therapeutic intervention strategy for KOA and KOA affected by abnormal mechanical loading, based on the causal evidence from our MR analysis.

Regarding the cascade regulatory pathway of IGFBP3-metabolite–KOA, 2-O-methylascorbic acid, a methylated derivative of vitamin C, may exert its mediating effect through 3 mechanisms: specific promotion of collagen synthesis, antioxidant activity, and reduction of cell apoptosis and proinflammatory cytokine expression.^[23,24]^ This metabolite maintains the activity of prolyl hydroxylase domain protein, thereby accelerating the hydroxylation modification of type II collagen α chains, which serve as the structural basis of the fibrous network in the cartilage matrix.^[25,26]^ In terms of antioxidation, 2-O-methylascorbic acid efficiently scavenges reactive oxygen species such as superoxide anions and hydroxyl radicals via its phenolic hydroxyl structure; it also regenerates glutathione (GSH) to synergistically maintain cellular redox homeostasis.^[27,28]^ For the regulation of cell fate and inflammation, this substance activates the PI3K/AKT pathway, inhibits the activation of NF-κB, and reduces the release of proinflammatory factors including tumor necrosis factor-α and interleukin [IL]-6.^[29]^ Okita K et al^[30]^demonstrated through in vitro cellular experiments that ascorbic acid positively regulates the expression of marker genes associated with collagen deposition, matrix formation, calcification, and chondrogenic differentiation by enhancing insulin-induced PI3K/AKT signaling transduction. Meacock S et al^[31]^ conducted experiments on mechanically induced osteoarthritis mice supplemented with multiple antioxidants including vitamin C, and found that mice fed an antioxidant-deficient control diet exhibited nearly twice the degree of osteoarthritis-related changes compared with the OA experimental group, with more pronounced disease progression. Although direct research on NAM in the pathogenesis of KOA to date is limited, studies on structurally similar substances provide indirect evidence. For instance, Lertratanangkoon K et al found that^[32]^ NAM can still exert a protective effect via alternative methyl donors (e.g., S-adenosylmethionine [SAMe]) even when GSH synthesis is inhibited, suggesting that its metabolites may exert indirect antioxidant effects through methylation pathways to alleviate KOA-related cartilage damage. Meanwhile, NAM enhances the supply of cysteine or GSH precursors, which in turn inhibits the release of proinflammatory factors such as tumor necrosis factor-α and IL-1*β*, alleviating joint inflammation and relieving pain and swelling in the context of KOA. Additionally, Yeh YT et al revealed that^[33]^ N-acetylcysteine (NAC) (a structural analog of NAM) significantly delays the progression of KOA in rats by reducing reactive oxygen species accumulation, inhibiting matrix metalloproteinase‑13 expression, and suppressing chondrocyte apoptosis. The levels of cartilage degradation markers (e.g., matrix metalloproteinase‑13) and inflammatory factors (e.g., IL-1*β*) were markedly decreased in the NAC-treated group. Furthermore, NAC can be converted into methionine, which is subsequently metabolized to SAMe; SAMe improves joint function by promoting collagen synthesis and inhibiting inflammation. As shown in a meta-analysis by Soeken KL et al, SAMe is comparable to nonsteroidal anti-inflammatory drugs in relieving osteoarthritis pain and improving joint function, but with fewer side effects.^[34]^ As the major binding protein of insulin‑like growth factor‑1 (IGF‑1), elevated plasma levels of IGFBP3 reduce the bioavailability of IGF-1.^[35]^ The traditional view holds that^[36]^ IGF-1 promotes the anabolic metabolism of chondrocytes via the PI3K/AKT pathway. However, the metabolite-mediated mechanism revealed in this study challenges this notion: instead of directly inhibiting the IGF-1 signaling pathway, IGFBP3 may indirectly suppress collagen synthesis and epigenetic regulation by reducing the levels of 2-O-methylascorbic acid and NAM. This finding explains why some clinical studies have demonstrated a positive correlation between IGFBP3 and KOA, which does not contradict the protective effect of IGF-1. Therefore, we can propose the hypothesis that the pathogenic effect of IGFBP3 may be mediated through metabolic intermediates rather than direct binding to IGF-1.

However, this study has several limitations that should be acknowledged. First, although MR analysis was conducted based on European population GWAS data and provided a methodological reference for KOA research in other populations through a “global data comparison + local validation” model, the application of international databases has significantly improved the efficiency of causal inference. However, attention should be paid to the effect heterogeneity that may be caused by genetic background differences. The generalizability of our findings to non-European populations requires further validation using population-specific GWAS data. Second, the exposure and outcome data were derived from different GWAS sources, and potential sample overlap may introduce bias. In addition, the mediation effect proportion for IGFBP3 was relatively low, suggesting the existence of other unidentified mediators or alternative pathways, and despite covering 1400 metabolites, the analysis did not include all biologically relevant metabolites. Third, while MR effectively addresses confounding and reverse causation, its findings represent indirect evidence for causal inference, necessitating further validation through basic experiments and animal models. In the field of KOA research, current studies still have many limitations: in research related to COL18A1, the cell-specific regulatory mechanism remains unclear, the functional differences of COL18A1 in different disease stages (such as the early inflammatory stage and the late cartilage degeneration stage) have not been thoroughly explored, and its interaction with other ECM regulatory factors (such as matrix metalloproteinases, transforming growth factor-*β*, etc) has not been clarified. For research on IGFBP3, there are problems such as a low mediation effect, insufficient validation of relevant metabolite data in other populations, and insufficient causal validation in animal models.

Therefore, future research can be carried out in several directions: at the basic mechanism level, verify the expression changes and functional differences of COL18A1 in different disease courses through in vivo animal models (such as constructing graded osteoarthritis models), and analyze the regulatory network between COL18A1 and other molecules by combining single-cell sequencing and protein-protein interaction analysis technologies; at the translational application level, exploring targeted drug design or genetic intervention strategies based on COL18A1 is also a promising research direction. For IGFBP3, it is necessary to first construct a regulatory network to explore new mediating factors, then carry out cohort studies in other populations to verify the specificity of the metabolic mediating effect, and finally use gene knockout mouse models to verify the causal relationship between metabolites and pathological progression. Both studies focus on molecular mechanisms and clinical transformation; in the future, efforts should be made to further integrate multi-omics technologies with in vitro and in vivo experiments, so as to provide a more solid theoretical basis and effective strategic guidance for the precise treatment of KOA.

## 5. Conclusion

This study was conducted around the plasma protein–plasma metabolite–KOA axis to dissect the causal associations among the 3 components. Collectively, our findings highlight that systematic interpretation integrating existing research evidence and practical clinical contexts is indispensable for an in-depth understanding of the study results. Specifically, MR analysis confirmed that human IGFBP3 exerts a protective effect against KOA by upregulating the levels of 2-O-methylascorbic acid and NAM, while COL18A1 was identified as another key factor that can attenuate KOA progression. These findings not only clarify the causal regulatory networks between plasma molecules and KOA but also provide novel molecular targets for the development of precise therapeutic strategies for KOA. Nevertheless, the limitations related to population heterogeneity and insufficient in vivo validation should be addressed in future studies to further translate these findings into clinical practice.

## Acknowledgments

The authors express their gratitude to all participants and researchers involved in this study. This study was supported by the National Natural Science Foundation of China (No. 82160913, led by Ping Zeng), the Guangxi Natural Science Foundation Project (No. 2025GXNSFAA069378, led by Xiang-Bin Rong), the Guangxi Medical and Health Appropriate Technology Development and Promotion Program (No. S2024102, led by Xiangbin Rong), the Guangxi Medical and Health Appropriate Technology Development and Promotion Program (No. GZSY2025034, led by Xiangbin Rong), and the Innovation Project of Guangxi Graduate Education of GXUCM (No. YCSY2025076, led by Haibo Zheng).

We sincerely thank the staff of the deCODE genetics database, the MRC IEU GWAS Summary Statistics Database, and all relevant institutions for providing the public datasets necessary for this study. In addition, we appreciate the technical support provided by the research team of Guangxi University of Chinese Medicine during the data analysis and manuscript preparation process. Furthermore, we would like to thank all researchers, technical personnel, and relevant assistants who have made contributions to this study but are not listed in the author list. Their efforts and support have provided an important guarantee for the smooth completion of this study.

## Author contributions

**Conceptualization:** Haibo Zheng, Xiangbin Rong.

**Data curation:** Haibo Zheng, Xianqiu Li, Longlong Wei, Xueshen Mo, Kun Hou.

**Formal analysis:** Haibo Zheng, Xianqiu Li, Hao Ni, Xueshen Mo, Kun Hou.

**Funding acquisition:** Haibo Zheng, Ping Zeng, Xiangbin Rong

**Investigation:** Haibo Zheng, Xueshen Mo, Kun Hou.

**Methodology:** Haibo Zheng, Xianqiu Li, Longlong Wei.

**Supervision:** Ping Zeng, Xiangbin Rong.

**Visualization:** Haibo Zheng, Xueshen Mo, Kun Hou.

**Writing – original draft:** Haibo Zheng, Xiangbin Rong.

**Writing – review & editing:** Haibo Zheng, Ping Zeng, Xiangbin Rong.


